# Dual Sensory Impairment as a Predictor of Loneliness and Isolation in Older Adults: National Cohort Study

**DOI:** 10.2196/39314

**Published:** 2022-11-14

**Authors:** Qiong Wang, Shimin Zhang, Yi Wang, Dan Zhao, Chengchao Zhou

**Affiliations:** 1 Centre for Health Management and Policy Research School of Public Health, Cheeloo College of Medicine Shandong University Jinan China; 2 National Health Commission of China Key Lab of Health Economics and Policy Research Shandong University Jinan China

**Keywords:** loneliness, social isolation, dual sensory impairment, vision impairment, hearing impairment, mental health

## Abstract

**Background:**

Loneliness and social isolation are global public health challenges. Sensory impairments (SIs) are highly prevalent among older adults but are often ignored as a part of normal aging. Identifying the role of SIs in loneliness and social isolation could provide insight into strategies for improving public health among older adults.

**Objective:**

This study aims to analyze the effects of SIs on loneliness and social isolation among older adults in rural and urban China.

**Methods:**

This cohort study of 3069 older adults (aged 60+) used data from 4 waves (2011, 2013, 2015, and 2018) of the China Health and Retirement Longitudinal Study (CHARLS), a nationally representative survey of adults aged 45 years or older. SIs include hearing impairment (HI), vision impairment (VI), and dual sensory impairment (DSI). DSI is defined as the co-occurrence of VI and HI. Participants with complete data on hearing, vision, social isolation, and loneliness were included in the analysis. Generalized estimating equation models adjusted for covariates were used to examine the relationships of DSI with loneliness and social isolation among older adults.

**Results:**

Older adults in rural areas have higher prevalence of DSI, loneliness, and social isolation than their urban counterparts. In rural areas, participants with VI only (odds ratio [OR] 1.34, 95% CI 1.12-1.62; *P*=.002), HI only (OR 1.32, 95% CI 1.02-1.71; *P*=.03), and DSI (OR 1.84, 95% CI 1.56-2.18; *P*<.001) were more likely to experience loneliness compared with participants without SIs. DSI showed a statistically significant association with loneliness compared with VI only (OR 1.37, 95% CI 1.22-1.54; *P*<.001) and HI only (OR 1.39, 95% CI 1.13-1.72; *P*=.002). In urban areas, participants with VI only (OR 2.44, 95% CI 1.57-3.80; *P*<.001), HI only (OR 2.47, 95% CI 1.41-4.32; *P*=.002), and DSI (OR 1.88, 95% CI 1.24-2.85; *P*=.003) were more likely to experience loneliness compared with participants without SIs. DSI was not associated with the increased likelihood of loneliness compared with HI only or VI only. SIs were not associated with social isolation among older adults in urban and rural areas. Until 2018, 86.97% (2669/3069) reported VI, but only 27.11% (832/3069) and 9.45% (290/3069) were treated with glasses and cataract surgery, respectively; besides, 75 individuals received both glasses and cataract surgery treatment. The prevalence of HI was 74.39% (2283/3069) in 2018, but only 0.72% (22/3069) were treated with a hearing aid.

**Conclusions:**

SIs are associated with an increased risk of loneliness rather than social isolation. A compounded risk of DSI on loneliness exists in rural areas rather than in urban areas. These findings expand our knowledge about the effects of SIs on loneliness and social isolation in non-Western populations. Interventions targeting HI only and DSI might be particularly effective for mitigating loneliness of older adults in urban and rural areas, respectively. Considering the high prevalence and low treatment rate of SIs, measures should be taken to make treatment more accessible.

## Introduction

Sensory impairments (SIs), comprising hearing impairment (HI), vision impairment (VI), and dual sensory impairment (DSI), are highly prevalent among older adults and increases with age [1]. The World Health Organization (WHO) reported that at least 2.2 billion people have VI or blindness [2]. It is estimated that approximately 1 in 10 people globally will have HI in 2050 [3]. With the increasing number of people with HI or VI, the prevalence of the co-occurrence of VI and HI, termed DSI, is expected to increase rapidly [4]. Previous studies have found that HI and VI have negative effects on mortality [5] and DSI worsens the effect of single SI [6]. Moreover, the negative effects of SIs on health outcomes among older adults are often overshadowed by the negative effects of chronic diseases and functional impairment [7,8]. The effects of SIs for older adults cannot be ignored and merit in-depth exploration.

SIs impose communication difficulties [9,10], difficulties with activities of daily living (ADL) [11], and decreased social participation [4,12], which may lead to impoverished social relationships, such as loneliness and social isolation. Loneliness is a subjective measure of an individual’s perceived discrepancy between desired and actual social interactions [13]. By contrast, social isolation refers to the objective state of estrangement, in which social connections are limited or absent [14]. Both loneliness and social isolation have become grand challenges of particular concerns for older adults given their independent association with a wide range of adverse health outcomes such as cognitive decline [15], depression [16,17], and mortality [14]. Reducing loneliness and social isolation in older adults is an important public health goal, which might be achieved by tackling modifiable risk factors or increasing social participation [18]. As a major obstacle of communication but a modifiable factor of aging, SIs merit more attention and addressing them may protect older adults against loneliness and social isolation.

Previous studies have examined the relationship between DSI and loneliness, but the results were inconsistent. For example, studies conducted in Western countries found an association between DSI and loneliness [19,20], whereas a study conducted in Malaysia did not support this association [21]. A paucity of research focused on the relationship between DSI and social isolation. Hajek and König [20] found a cross-sectional association between DSI and social isolation among Germans aged 40 and older. However, there is a complete lack of studies investigating the longitudinal relationship between DSI and social isolation. Given the geographic, racial, and cultural differences between Western and Asian populations, the relationships of DSI with loneliness and social isolation merit further research based on local conditions. China is changing rapidly in population aging and internal migration [22]. Awareness of DSI and its impact on loneliness and social isolation may be important to help Chinese older adults maintain a good quality of life and promote healthy aging. Moreover, due to great disparity existing in socioeconomic status and health care resources between rural and urban areas [23], older adults in rural areas might be at a higher risk of loneliness, social isolation, and DSI than those in urban areas. Thus, it is necessary to stratify the analyses by region of residence in this study.

To our knowledge, the longitudinal relationships of DSI with loneliness and social isolation in Chinese older adults have not been studied. Moreover, the relative relationship between older adults with DSI and those with single SI was less clear. In addition, whether the effect of DSI on loneliness and social isolation among older adults is similar between rural and urban areas remains unclear. Therefore, this study aims to assess the longitudinal relationships of DSI with loneliness and social isolation and examine whether these associations differ in rural and urban China.

## Methods

### Participants

The data used in this study were from the 2011, 2013, 2015, and 2018 waves of the China Health and Retirement Longitudinal Study (CHARLS). It is a nationally representative longitudinal study that surveys Chinese residents aged 45 years or older since 2011 (wave 1). It covers not only personal information and environmental information, but also factual information and attitude information, such as sociodemographic characteristics, socioeconomic status, health status, and psychological conditions. In the sampling method, a stratified (by per capita GDP of urban districts and rural counties) multistage (county/district-village/community household) random sampling strategy was adopted, and finally a total of 150 counties in 28 provinces of China were sampled [24]. The CHARLS baseline survey in 2011 included 17,708 respondents aged 45 years or older. Up to wave 4, 5587 respondents were lost to follow-up. For this study, we excluded older adults aged less than 60 years (n=7217) at baseline. Then, older adults with missing data (n=987) on main variables (SIs, loneliness, and social isolation) and who moved between urban and rural areas during follow-up (n=848) were excluded. Finally, a total of 3069 older adults who participated in all follow-up waves were included in our study, with 530 urban residents and 2539 rural residents (Multimedia Appendix 1).

### Measures

#### Loneliness

Loneliness was measured by a single item of the Center for Epidemiological Studies Depression Scale (CESD), “In the last week, how often did you feel lonely?” [25]. This single measurement is highly correlated with multi-item loneliness scales, such as the University of California Los Angeles Loneliness Scale, and has been used in many previous studies [26,27]. Based on previous research experience [25,26], respondents were considered lonely if they feel lonely on some days (1-2 days), occasionally (3-4 days), or most of the time (5-7 days). Respondents were considered not lonely if they feel lonely rarely or none of the time (<1 day).

#### Social Isolation

We created an index of social isolation by giving 1 point for each of being unmarried, living alone, having less than weekly contact (by phone, in person, or by email) with children, and not participating in any social activities over the last month (eg, interacted with friends; played chess or cards; went to sport, social, or other clubs). Scores ranged from 0 to 4, with higher scores indicating a higher level of social isolation. Because of the positively skewed distribution of social isolation scores, we categorized participants according to the top quintile (>1 for social isolation) [28].

#### Sensory Impairments

The self-reported data on VI were composed of 2 questions: (1) “How good is your vision for seeing things at a distance (with glasses or corrective lenses), like recognizing a friend from across the street?” and (2) “How good is your vision for seeing things up close (with glasses or corrective lenses), like reading ordinary newspaper print?” For each question, the responses included “excellent,” “very good,” “good,” “fair,” or “poor.” We identified respondents as having VI if they reported fair or poor vision (for either long distance or near vision). One question was used to assess HI, “Is your hearing excellent, very good, good, fair, or poor (with a hearing aid if you normally use it and without if you normally don’t).” Participants were identified as having HI if they reported fair or poor hearing. When HI and VI were both present, participants were regarded as having DSI. SIs assessment and categorization was in accordance with previous studies [29,30].

#### Covariates

Sociodemographic characteristics, lifestyle factors, and health-related variables were considered as potential confounding variables. Demographic characteristics included gender (male/female), age (mean and SD), educational level (lower than primary school/primary school/middle school, or above), and household income. The household total annual income is the sum of all income at the household level including that from earning income, capital income, pension income, income from government transfers, other income, and the total income from other household members (eg, from parents, children, relatives). Lifestyle factors included smoking status (yes/no) and alcohol drinking (yes/no). Health-related variables were collected by asking their chronic health conditions and functional impairment. We categorized the self-reported chronic diseases into 3 groups (no chronic disease/1 chronic disease/multimorbidity). Functional impairment was assessed using the ADL scale which consists of 6 items including dressing, bathing or showering, eating, getting in or out of bed, toileting, and controlling urination and defecation. The ADL score ranges from 6 to 24, with a higher score indicating the worse ability of daily living activities [31].

### Statistical Analysis

Descriptive statistics were used to describe the baseline characteristics of the sample. Continuous variables were summarized using means and SDs. Categorical variables were reported using numbers and percentages. The generalized estimating equation model assuming an independent working correlation structure was used to examine the associations of SIs with loneliness and social isolation among older adults in rural and urban areas during the follow-up period. We calculated the estimate odds ratios (ORs) and 95% CIs while adjusting for all identified confounders. Furthermore, in sensitivity analysis, we treated social isolation as a continuous variable to assess the robustness of the relationship between SIs and social isolation. A 2-sided *P*<.05 was considered statistically significant. Stata version 14.2 (StataCorp) was used for the data analyses.

### Ethical Considerations

This study protocol was approved and organized by Peking University’s Institutional Review Board (IRB00001052-11015). All procedures were in accordance with the 1964 Declaration of Helsinki and its later amendments or comparable ethical standards. This survey was anonymous, and the answers were protected by privacy law. Written informed consents clarifying the study purposes were obtained from each participant before the survey.

## Results

Tables 1 and 2 show the baseline characteristics of the urban and rural respondents, respectively. Mean (SD) age of the 3069 respondents was 66.02 (5.15) years, and 51.28% (1574/3069) were female. Of all participants, being more socially isolated or lonelier was associated with being older, being female, less educated, lower income, being a smoker, a higher level of functional impairment, more kinds of chronic diseases, and having 1 of the SIs. Until 2018, 83.8% (444/530) reported VI in urban areas, 45.5% (241/530) were treated with glasses, and 10.6% (56/530) were treated with cataract surgery. The prevalence of VI is 87.63% (2225/2539) in rural areas, but only 23.28% (591/2539) and 9.22% (234/2539) were treated with glasses and cataract surgery, respectively. Until 2018, 70% (371/530) and 75.31% (1912/2539) reported HI in urban and rural areas, but only 1.13% (6/530) and 0.63% (16/2539) were treated with a hearing aid, respectively (Multimedia Appendix 2).

Table 3 displays the prevalence of loneliness, social isolation, and SIs over time in urban and rural areas, respectively. The prevalence rates of loneliness, social isolation, and DSI are on the rise overall. At baseline (2011), 5.60% (172/3069) reported HI only, 25.48% (782/3069) reported VI only, and 54.90% (1685/3069) reported DSI. Participants in rural areas were more likely to report DSI (1442/2539, 56.79% vs 243/530, 45.85%; *P*<.001) and loneliness (847/2539, 33.36% vs 116/530, 21.89%; *P*<.001) than those in urban areas. There was no statistical difference in the prevalence of social isolation among urban and rural older adults at baseline. In 2018, the prevalence of social isolation in rural areas was higher than that in urban areas (739/2539, 29.11% vs 125/530, 23.58%; *P*=.01).

**Table 1:** Baseline characteristics of older adults in urban China (n=530).

		No loneliness	Loneliness	*P* value^a^	Low social isolation	High social isolation		*P* value^a^
**Gender, n (%^b^)**				*.03*				*<.001*
	Male	215 (82.06)	47 (17.94)		237 (90.46)		25 (9.54)	
	Female	199 (74.25)	69 (25.75)		203 (75.75)		65 (24.25)	
Age, mean (SD)		66.37 (5.54)	66.67 (5.37)	.92	66.09 (5.11)	69.80 (6.25)		*<.001*
**Education, n (%)**				*.005*				*<.001*
	Lower than primary school	40 (68.97)	18 (31.03)		38 (65.52)		20 (34.48)	
	Primary school	141 (73.06)	52 (26.94)		154 (79.79)		39 (20.21)	
	Middle school or above	233 (83.51)	46 (16.49)		248 (88.89)		31 (11.11)	
**Marital status, n (%)**				*<.001*				—^c^
	Couple	357 (82.07)	78 (17.93)		413 (94.94)		22 (5.06)	
	Single	57 (60.00)	38 (40.00)		27 (28.42)		68 (71.58)	
**Household annual income, n (%)^d^**				*<.001*				*<.001*
	Q1	85 (63.91)	48 (36.09)		80 (60.15)		53 (39.85)	
	Q2	176 (78.92)	47 (21.08)		193 (86.55)		30 (13.45)	
	Q3	39 (92.86)	3 (7.14)		40 (95.24)		2 (4.76)	
	Q4	114 (86.36)	18 (13.64)		127 (96.21)		5 (3.79)	
**Smoking status, n (%)**				.52				*.047*
	No	329 (78.71)	89 (21.29)		340 (81.34)		78 (18.66)	
	Yes	85 (75.89)	27 (24.11)		100 (89.29)		12 (10.71)	
**Alcohol consumption, n (%)**				.17				*.02*
	No	287 (76.53)	88 (23.47)		302 (80.53)		73 (19.47)	
	Yes	127 (81.94)	28 (18.06)		138 (89.03)		17 (10.97)	
Activities of daily living, mean (SD)		6.24 (1.16)	6.33 (0.84)	.45	6.23 (0.95)	6.39 (1.65)		.22
**Chronic disease, n (%)**				*.045*				.78
	None	103 (83.06)	21 (16.94)		101 (81.45)		23 (18.55)	
	One	108 (82.44)	23 (17.56)		111 (84.73)		20 (15.27)	
	≥2	203 (73.82)	72 (26.18)		228 (82.91)		47 (17.09)	
**Sensory impairments, n (%)**				*.01*				.77
	No sensory impairments	80 (88.89)	10 (11.11)		73 (81.11)		17 (18.89)	
	Hearing impairment only	31 (83.78)	6 (16.22)		31 (83.78)		6 (16.22)	
	Vision impairment only	114 (71.25)	46 (28.75)		130 (81.25)		30 (18.75)	
	Dual sensory impairment	189 (77.78)	54 (22.22)		206 (84.77)		37 (15.23)	

^a^Italicized values denote statistical significance (*P*<.05) between the groups.

^b^Percentages were estimated over cases with valid data in every group.

^c^Chi-square test was not performed.

^d^Q1 was the poorest and Q4 was the richest. Q1: ≦US $415; Q2: US $416-1981; Q3: US $1982-3391; Q4: >US $3391.

**Table 2:** Baseline characteristics of older adults in rural China (n=2539).

			No loneliness		Loneliness		*P* value^a^		Low social isolation		High social isolation		*P* value^a^
**Gender, n (%^b^)**							*<.001*						*.003*
	Male	879 (71.29)		354 (28.71)				1038 (84.18)		195 (15.82)			
	Female	813 (62.25)		493 (37.75)				1041 (79.71)		265 (20.29)			
Age, mean (SD)			65.69 (5.00)		66.26 (5.18)		*.008*		65.50 (4.83)		67.60 (5.72)		*<.001*
**Education, n (%)**							*<.001*						*<.001*
	Lower than primary school	695 (62.39)		419 (37.61)				873 (78.37)		241 (21.63)			
	Primary school	809 (69.15)		361 (30.85)				990 (84.62)		180 (15.38)			
	Middle school or above	188 (73.73)		67 (26.27)				216 (84.71)		39 (15.29)			
**Marital status, n (%)**							*<.001*						—^c^
	Couple	1504 (70.98)		615 (29.02)				1968 (92.87)		151 (7.13)			
	Single	188 (44.76)		232 (55.24)				111 (26.43)		309 (73.57)			
**Household annual income, n (%)^d^**							*.001*						*<.001*
	Q1	412 (64.88)		223 (35.12)				446 (70.24)		189 (29.76)			
	Q2	390 (61.42)		245 (38.58)				504 (79.37)		131 (20.63)			
	Q3	508 (69.59)		222 (30.41)				635 (86.99)		95 (13.01)			
	Q4	382 (70.87)		157 (29.13)				494 (91.65)		45 (8.35)			
**Smoking status, n (%)**							.32						.34
	No	1119 (65.98)		577 (34.02)				1380 (81.37)		316 (18.63)			
	Yes	573 (67.97)		270 (32.03)				699 (82.92)		144 (17.08)			
**Alcohol consumption, n (%)**							.87						.52
	No	1139 (66.53)		573 (33.47)				1396 (81.54)		316 (18.46)			
	Yes	553 (66.87)		274 (33.13)				683 (82.59)		144 (17.41)			
Activities of daily living, mean (SD)			6.41 (1.16)		6.81 (1.68)		*<.001*		6.53 (1.37)		6.59 (1.35)		.40
**Chronic disease, n (%)**							*<.001*						.40
	None	494 (72.43)		188 (27.57)				563 (82.55)		119 (17.45)			
	One	547 (70.13)		233 (29.87)				647 (82.95)		133 (17.05)			
	≥2	651 (60.45)		426 (39.55)				869 (80.69)		208 (19.31)			
**Sensory impairments, n (%)**							*<.001*						.09
	No sensory impairments	264 (77.65)		76 (22.35)				277 (81.47)		63 (18.53)			
	Hearing impairment only	100 (74.07)		35 (25.93)				100 (74.07)		35 (25.93)			
	Vision impairment only	441 (70.90)		181 (29.10)				507 (81.51)		115 (18.49)			
	Dual sensory impairment	887 (61.51)		555 (38.49)				1195 (82.87)		247 (17.13)			

^a^Italicized values indicate statistical significance (*P*<.05) between the groups.

^b^Percentages were estimated over cases with valid data in every group.

^c^Chi-square test was not performed.

^d^Q1 was the poorest and Q4 was the richest. Q1: ≦US $415; Q2: US $416-1981; Q3: US $1982-3391; Q4: >US $3391.

**Table 3:** The prevalence of loneliness, social isolation, and SIs^a^ in urban and rural China (N=3069).

Setting			2011, n (%)	2013, n (%)	2015, n (%)	2018, n (%)
**Urban areas (n=530)**						
	**Loneliness**					
		No	414 (78.11)	441 (83.21)	429 (80.94)	402 (75.85)
		Yes	116 (21.89)	89 (16.79)	101 (19.06)	128 (24.15)
	**Social isolation**					
		Low	440 (83.02)	455 (85.85)	440 (83.02)	405 (76.42)
		High	90 (16.98)	75 (14.15)	90 (16.98)	125 (23.58)
	**SIs**					
		No SIs	90 (16.98)	74 (13.96)	65 (12.26)	56 (10.57)
		HI^b^ only	37 (6.98)	55 (10.38)	32 (6.04)	30 (5.66)
		VI^c^ only	160 (30.19)	131 (24.72)	121 (22.83)	103 (19.43)
		DSI^d^	243 (45.85)	270 (50.94)	312 (58.87)	341 (64.34)
**Rural areas (n=2539)**						
	**Loneliness**					
		No	1692 (66.64)	1876 (73.89)	1732 (68.22)	1584 (62.39)
		Yes	847 (33.36)^e,f^	663 (26.11)^e,f^	807 (31.78)^e,f^	955 (37.61)^e,f^
	**Social isolation**					
		Low	2079 (81.88)	2071 (81.57)	2023 (79.68)	1800 (70.89)
		High	460 (18.12)^e^	468 (18.43)^e,g^	516 (20.32)^e^	739 (29.11)^e,g^
	**SIs**					
		No SIs	340 (13.39)	284 (11.19)	207 (8.15)	209 (8.23)
		HI only	135 (5.32)	163 (6.42)	123 (4.84)	105 (4.14)
		VI only	622 (24.50)	568 (22.37)	460 (18.12)	418 (16.46)
		DSI	1442 (56.79)^e,f^	1524 (60.02)^e,f^	1749 (68.89)^e,f^	1807 (71.17)^e,g^

^a^SI: sensory impairments.

^b^HI: hearing impairment.

^c^VI: vision impairment.

^d^DSI: dual sensory impairment.

^e^Compared with urban areas.

^f^*P*<.001.

^g^*P*<.05.

**Figure 1:**
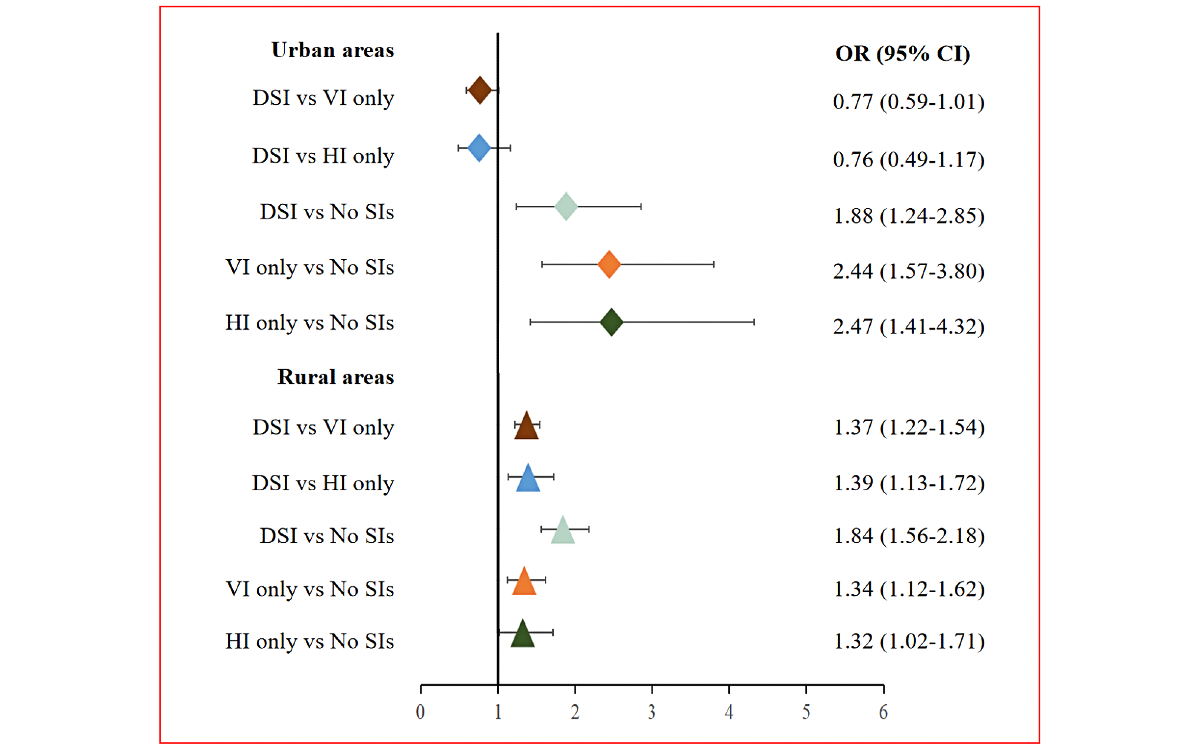
Association between SIs and loneliness among older adults in urban and rural China. DSI: dual sensory impairment; HI: hearing impairment; SIs: sensory impairments; VI: vision impairment.

**Figure 2:**
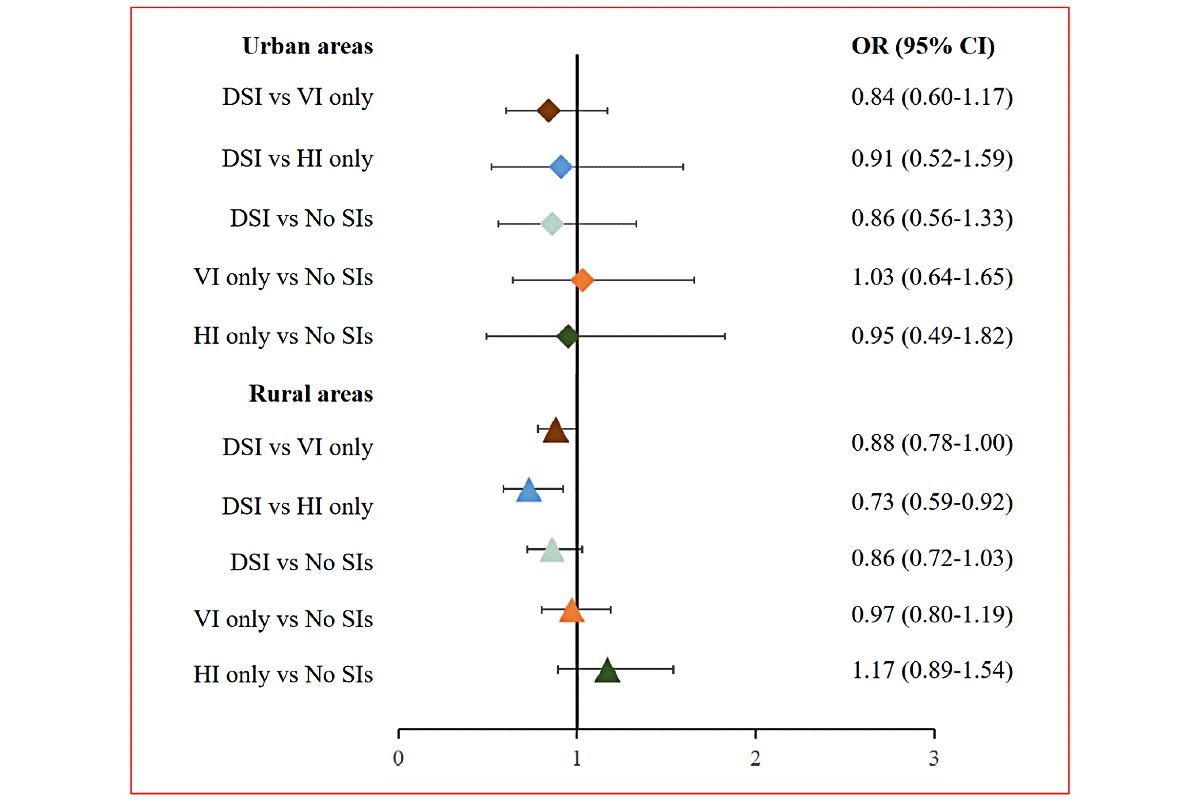
Association between SIs and social isolation among older adults in urban and rural China. DSI: dual sensory impairment; HI: hearing impairment; SIs: sensory impairments; VI: vision impairment.

As shown in Figure 1, among older adults in urban areas, participants with VI only (OR 2.44, 95% CI 1.57-3.80; *P*<.001), HI only (OR 2.47, 95% CI 1.41-4.32; *P*=.002), and DSI (OR 1.88, 95% CI 1.24-2.85; *P*=.003) were more likely to experience loneliness compared with participants without SIs. Compared with HI only (OR 0.76, 95% CI 0.49-1.17; *P*=.22) or VI only (OR 0.77, 95% CI 0.59-1.01; *P*=.06), DSI showed an insignificant association with loneliness. Among older adults in rural areas, participants with DSI were more likely to experience loneliness compared with those without SIs (OR 1.84, 95% CI 1.56-2.18; *P*<.001), those with VI only (OR 1.37, 95% CI 1.22-1.54; *P*<.001), and HI only (OR 1.39, 95% CI 1.13-1.72; *P*=.002). Both VI only (OR 1.34, 95% CI 1.12-1.62; *P*=.002) and HI only (OR 1.32, 95% CI 1.02-1.71; *P*=.03) were associated with increased feelings of loneliness. Regardless of the rural or urban location, the effects of SIs on social isolation were not statistically significant (urban areas: DSI vs no SIs: *P*=.50; VI only vs no SIs: *P*=.92; HI only vs no SIs: *P*=.87; rural areas: DSI vs no SIs: *P*=.10; VI only vs no SIs: *P*=.80; HI only vs no SIs: *P*=.25; Figure 2). The results of the sensitivity analysis were consistent with the main analysis (Multimedia Appendix 3).

## Discussion

### Principal Findings

Using longitudinal data from a nationally representative sample, this study provides an insight into the effect of DSI on loneliness and social isolation among Chinese older adults living in urban and rural areas. Results show that HI only, VI only, and DSI are each independently associated with a higher risk of loneliness in both urban and rural areas. HI and VI have a synergistic relationship with loneliness in rural areas, but we did not observe such an effect in urban areas. Our findings supported the view that SIs did not affect social isolation.

There are multiple reasons why older adults in rural areas have higher prevalence of DSI, loneliness, and social isolation than their urban counterparts. Older adults in rural areas may rarely receive appropriate treatment measures when HI or VI appears, as they have fewer health care resources, lower access to several health information sources, and poorer socioeconomic status compared with their urban counterparts [32-34]. With rapid industrialization and urbanization, there is massive migration of younger and middle-aged people from rural to urban areas, leaving older adults to either live alone or with their spouse in rural areas. Older adults with SIs who lived in rural areas had poorer perceived tangible support than their urban counterparts [35]. Furthermore, public services and voluntary organizations in rural areas are less developed than those in urban areas; consequently, older adults in rural areas have less choices for participating in social activities [36]. Therefore, older adults in rural areas might be more likely to experience loneliness and social isolation.

Our study found that individuals with SIs experience more loneliness compared with those without SIs, which was consistent with other studies [19,37]. Older adults with SIs are often lonely but they are not necessarily socially isolated. One possible reason is that SIs influence loneliness by affecting communication with their closest relatives or friends, as loneliness is not caused by being alone but rather by the unmet affective gain of their closest relationship [38]. Previous studies have indicated that older adults with DSI were more prone to experiencing a breakdown in communication compared with those without SIs [39,40]. Older adults with HI may feel frustrated or embarrassed over their difficulty communicating, resulting in loneliness [41]. Another reason may be neural changes associated with SIs. HI may contribute to changes in the frontal lobe, which alter the regulation of emotion [42], that may contribute to the likelihood of loneliness.

Surprisingly, older adults with DSI are at a compounded risk of loneliness compared with those with HI only or VI only in rural areas, but not in urban areas. Previous studies reported similar results, but there was no difference in mental health between older adults with DSI and single SI [43-45]; however, these studies did not consider the regional difference. A typical cultural and personal value among Chinese older adults was that they do not tend to bother others or even their adult children as they perceived that people are very busy with their own lives; therefore, they scarcely bother to seek help even though they may be sick [46]. In other words, older adults almost do not depend on their children until they are unable to take care of themselves, although children are the main caregivers for their older parents in China. Older adults with HI only or VI only were able to compensate for their impairment in one sense with the other so that they could take care of themselves to some extent [37]. As a particularly vulnerable group with challenges, older adults with DSI probably have to count on their children [47]. In urban areas, older adults with DSI could get more attention and care from their children in a timely manner, which might help them relieve loneliness. Likewise, older adults with DSI also deserve more attention and care in rural areas. However, most of the rural adults out-migrate to the cities for work and cannot take care of their parents by their side. Phone calls appear to be the main way of communication between older adults and their children, but this may be limited by HI unfortunately. Moreover, older parents with DSI have to reduce social activities due to the lack of their caregivers’ help. Consequently, older parents with DSI who lived in rural areas may be at a higher risk to be lonely.

Previous cross-sectional studies have yielded conflicting results on the relationship between single SI and social isolation. A systematic review indicated that most studies found a relationship between HI and social isolation [41]. Kotwal et al [48] also found that HI rather than VI was associated with social isolation among older adults. However, significant correlations were found between VI and social isolation in another study [20]. Interestingly, in this longitudinal study, social isolation of older adults with SIs were not fundamentally different from those without SIs. According to the Socioemotional Selectivity Theory, older adults may selectively narrow their range of social partners and focus more on their closest relationships [38]. Reduction in social networks is likely normal in older adults, regardless of their health status including SIs and ADL limitation. Notably, undesired social isolation represents a low quality of social relationships and is very closely related to loneliness [49,50]. This led us to speculate that SIs may affect undesired social isolation. Future research is needed to shed more light on this issue. In addition, given the high prevalence of social isolation found in this study, observation should continuously be made for any signs of negative health outcomes in older adults with social isolation. Besides, active steps should be taken to prevent older adults from being socially isolated. Maintaining ties with family members and friends is important for preventing social isolation among American older adults [50]. In the context of Chinese culture, social ties to children may be an important priority in older adults.

### Implications and Contribution

Our findings provide some new inspiration for older adults to relieve loneliness. Many prior interventions were conducted to mitigate loneliness by stimulating socialization [51], but they rarely considered that SIs may prevent older adults from enrolling or adhering to an intervention. Furthermore, an individual may be lonely without being socially isolated [49]. Future interventions considering SIs might be more accessible and effective for reducing loneliness among older adults. Recently, efforts to improve hearing have already shown beneficial effects on loneliness [52,53]. However, 74.39% (2283/3069) of older adults reported HI in 2018, but only 0.72% (22/3069) were treated with a hearing aid in this study. This study emphasized that preventing HI among older adults is of high priority to reduce their risk of loneliness. In addition, we propose that parents with single SI in urban areas and those with DSI in rural areas should be get more attention from their children.

### Strengths and Limitations

A particular strength of this study is the longitudinal examination of the effects of DSI on loneliness and social isolation by region of residence in Chinese older adults. However, several limitations of this study should be noted. First, SIs were self-reported, which might result in some bias. Participants may overestimate or underestimate their abilities to see and hear. Further research should use clinical diagnostic measurements to verify the data. Second, due to data limitation, the severity of SIs was not measured. Although the prevalence of SIs in urban areas was lower than that in rural areas, whether the severity of SIs in urban areas is higher than that in rural areas remains unknown, as urban people are more likely to experience occupational noise exposure. Future studies are therefore needed to consider these issues as the more serious SIs older adults have, the more likely they are to have physical and mental health problems [37,54]. Finally, loneliness was assessed with only 1 question. Although this measure was widely used in the literature [25,26], it might be less reliable than a composite measure.

### Conclusion

Overall, this study found that SIs were significantly associated with loneliness rather than social isolation among older adults living in both urban and rural China. A synergistic effect of HI and VI on loneliness was observed in rural areas, but such an effect was not found in urban areas. A better understanding of the longitudinal effect of SIs on loneliness by region of residence could help policy makers to allocate health resources and conduct targeted interventions accordingly.

## Appendix

Multimedia Appendix 1 The flow chart of study respondent.

Multimedia Appendix 2 The prevalence of loneliness and social isolation in treated and untreated participants in 2018.

Multimedia Appendix 3 Association between SIs and social isolation among older adults in urban and rural China.

## Abbreviations

ADL: activities of daily living
CESD: Center for Epidemiological Studies Depression Scale
CHARLS: China Health and Retirement Longitudinal Study
DSI: dual sensory impairment
HI: hearing impairment
SI: sensory impairment
VI: vision impairment
OR: odds ratio
WHO: World Health Organization

## Data Availability

The original data sets are publicly available at [55]. The data sets generated during and analyzed during this study are available from the corresponding author on reasonable request.
