# Supplementary figures and images for: Dual Sensory Impairment as a Predictor of Loneliness and Isolation in Older Adults: National Cohort Study
Source: JMIR Public Health Surveill. 2022 Nov 14;8(11):e39314. doi: 10.2196/39314 (PMC9706378; doi:10.2196/39314)

**Multimedia Appendix 1.**

**Figure S1.** The flow chart of study respondent.


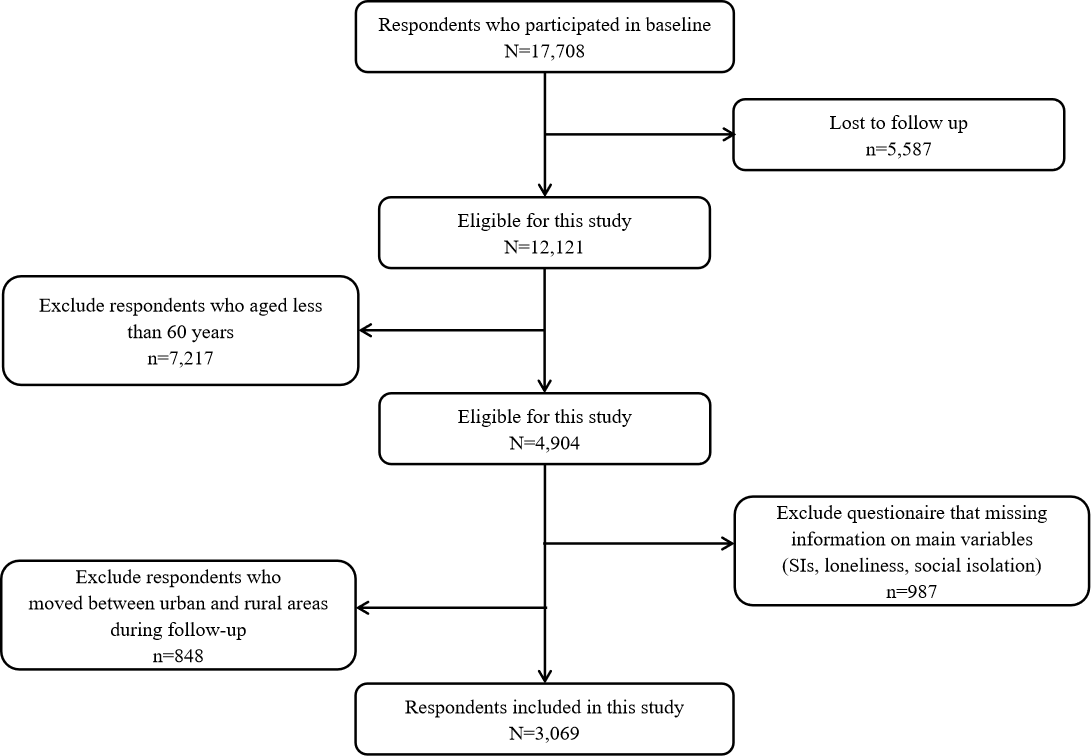

Supplement: Multimedia Appendix 1 [file publichealth_v8i11e39314_app1.docx]
