# Supplementary material for: Dual Sensory Impairment as a Predictor of Loneliness and Isolation in Older Adults: National Cohort Study
Source: JMIR Public Health Surveill. 2022 Nov 14;8(11):e39314. doi: 10.2196/39314 (PMC9706378; doi:10.2196/39314)
Supplement: Multimedia Appendix 2 [file publichealth_v8i11e39314_app2.docx]

**Multimedia Appendix 2.** The prevalence of loneliness and social isolation in treated and untreated participants in 2018.

|  | | No loneliness n (%) | Loneliness n (%) | *P* value | Low social isolation n (%) | High social isolation n (%) | *P* value |
| --- | --- | --- | --- | --- | --- | --- | --- |
| Urban areas |  |  |  |  |  |  |  |
| No VI | Untreated | 39 (79.59) | 10 (20.41) | .21 | 39 (79.59) | 10 (20.41) | .32 |
|  | Treated | 25 (67.57) | 12 (32.43) |  | 26 (70.27) | 11 (29.73) |  |
| VI | Untreated | 156 (75.36) | 51 (24.64) | .72 | 157 (75.85) | 50 (24.15) | .73 |
|  | Treated | 182 (76.79) | 55 (23.21) |  | 183 (77.22) | 54 (22.78) |  |
| No HI | Untreated | 128 (80.50) | 31 (19.50) | - | 128 (80.50) | 31 (19.50) | - |
|  | Treated | 0 | 0 |  | 0 | 0 |  |
| HI | Untreated | 271 (74.25) | 94 (25.75) | - | 272 (74.52) | 93 (25.48) | - |
|  | Treated | 3 (50.00) | 3 (50.00) |  | 5 (83.33) | 1 (16.67) |  |
| Rural areas |  |  |  |  |  |  |  |
| No VI | Untreated | 156 (66.95) | 77 (33.05) | .11 | 155 (66.25) | 78 (33.48) | .09 |
|  | Treated | 62 (76.54) | 19 (23.46) |  | 62 (76.54) | 19 (23.46) |  |
| VI | Untreated | 939 (61.25) | 594 (38.75) | .84 | 1087 (70.91) | 446 (29.09) | .71 |
|  | Treated | 427 (61.71) | 265 (38.29) |  | 496 (71.68) | 196 (28.32) |  |
| No HI | Untreated | 438 (70.08) | 187 (29.92) | - | 437 (69.92) | 188 (30.08) | - |
|  | Treated | 1 (50.00) | 1 (50.00) |  | 1 (50.00) | 1 (50.00) |  |
| HI | Untreated | 1136 (59.85) | 762 (40.15) | - | 1353 (71.29) | 545 (28.71) | - |
|  | Treated | 9 (64.29) | 5 (35.71) |  | 9 (64.29) | 5 (35.71) |  |

*Notes:*  HI = Hearing Impairment; VI = Vision Impairment. Treated: VI was treated with glasses or cataract surgery; HI was treated with hearing aid.
