# Supplementary material for: Dual Sensory Impairment as a Predictor of Loneliness and Isolation in Older Adults: National Cohort Study
Source: JMIR Public Health Surveill. 2022 Nov 14;8(11):e39314. doi: 10.2196/39314 (PMC9706378; doi:10.2196/39314)
Supplement: Multimedia Appendix 3 [file publichealth_v8i11e39314_app3.docx]

**Multimedia Appendix 3.** Association between SIs and social isolation among older adults in urban and rural China.

|  | Urban areas | | Rural areas | |
| --- | --- | --- | --- | --- |
|  | β | 95% CI | β | 95% CI |
| HI only vs. No SIs | 0.014 | (-0.05,0.08) | 0.025 | (-0.01,0.06) |
| VI only vs. No SIs | 0.022 | (-0.02,0.07) | -0.016 | (-0.04,0.01) |
| DSI vs. No SIs | 0.039 | (-0.01,0.09) | -0.010 | (-0.04,0.02) |
| DSI vs. HI only | 0.026 | (-0.03,0.08) | -0.035 | (-0.07,-0.002) |
| DSI vs. VI only | 0.017 | (-0.02,0.05) | 0.006 | (-0.01,0.03) |

*Notes:* Models were adjusted for gender, age, education, household income, smoking status, alcohol drinking status, functional impairment, chronic disease, loneliness, and follow-up time. CI = confidence interval; SIs = Sensory Impairments; HI only = Hearing Impairment only; VI only = Vision Impairment only; DSI = Dual Sensory Impairment.
